# Reduction in postpartum weight with laser acupuncture: A randomized control trial

**DOI:** 10.1097/MD.0000000000004716

**Published:** 2016-08-26

**Authors:** Yu-Chiang Hung, I-Ling Hung, Wen-Long Hu, Ying-Jung Tseng, Chun-En Kuo, Yen-Nung Liao, Bei-Yu Wu, Ching-Chang Tsai, Pei-Yuan Tsai, Hsin-Ping Chen, Meng-Hsuan Huang, Fang-Yen Su

**Affiliations:** a Department of Chinese Medicine; b Department of Gynecology and Obstetrics; c Kaohsiung Chang Gung Memorial Hospital and Chang Gung University College of Medicine; d School of Chinese Medicine for Post Baccalaureate, I-Shou University; e Fooyin University College of Nursing; f Kaohsiung Medical University College of Medicine, Kaohsiung, Taiwan.

**Keywords:** laser acupuncture, obesity, postpartum weight retention, traditional Chinese medicine

## Abstract

**Background:**

Gestational weight gain and weight retention at 1 year after delivery are associated with long-term obesity. We aimed to investigate the effect of laser acupuncture therapy on postpartum weight control.

**Methods:**

We randomly assigned 66 subjects with postpartum weight retention to a laser acupuncture group and control group. The subjects were treated at acupoints including the stomach and hunger points of the ear, ST25, ST28, ST40, SP15, CV9, and SP6 by using verum or sham laser acupuncture over 5 sessions per week. After 12 treatment sessions, the differences in the body mass index (BMI), body fat percentage (BFP), and waist-to-buttocks ratio (WBR) of the patients were analyzed and compared between the laser acupuncture and control groups via analysis of variance, chi-square tests, and stepwise regression tests.

**Results:**

The characteristics of the patients did not significantly differ between the laser acupuncture and control groups. Analysis of repeated measures data between the laser acupuncture and control groups indicated the presence of significant differences in postpartum BMI (*P* < 0.001) and BFP (*P* < 0.001); however, no significant difference was observed for WBR (*P* = 0.09).

**Conclusion:**

Laser acupuncture reduces postpartum weight retention by improving BMI and BFP, but does not impact the WBR following short-term treatment.

## 1 Introduction

Obesity is an important healthcare issue worldwide.^[1–3]^ It is associated with increased risks of diabetes mellitus, hypertension, and other cardiometabolic complications, and can hence increase the burden on medical care. The recent increases in the rates of overweight and obesity appear to be more pronounced in women.^[4]^ High gestational weight gain (GWG) would be an important predictor factor for the afterward birth weight.^[5]^ In particular, pregnancy-related weight gain has been identified as a potentially major contributor to higher rates of overweight and obesity in women of reproductive age.^[6]^ In fact, postpartum weight retention is a significant contributor to the risk for obesity in women at 1 year after delivery, even among women with normal weight before pregnancy.^[7]^ Some studies have indicated that GWG (weight gained during pregnancy) and weight retention at 1 year after delivery are associated with long-term obesity.^[8,9]^ Therefore, several attempts are being made to identify effective methods for postpartum weight control.

The conventional therapy for obesity includes the consumption of a low-calorie diet, following a certain exercise regimen and treatment with different types of slimming drugs. Traditional Chinese medicine (TCM), including herbal medication and acupuncture, may also aid in body weight control. In particular, laser acupuncture therapy (LAT) has been used for the treatment of several conditions, including obesity.^[10]^ Our previous study examined the effects of LAT in the treatment of simple obesity, whereas Wozniak et al focused on postmenopausal women with visceral obesity.^[11,12]^ To our knowledge, no study has assessed the use of LAT for postpartum weight control. In the present study, we aimed to investigate the effects of LAT on postpartum weight control, based on the TCM theory, which advocates treatment of the ear and body acupoints to modulate metabolism.

## 2 Methods

### 2.1 Participants

**Figure 1:**
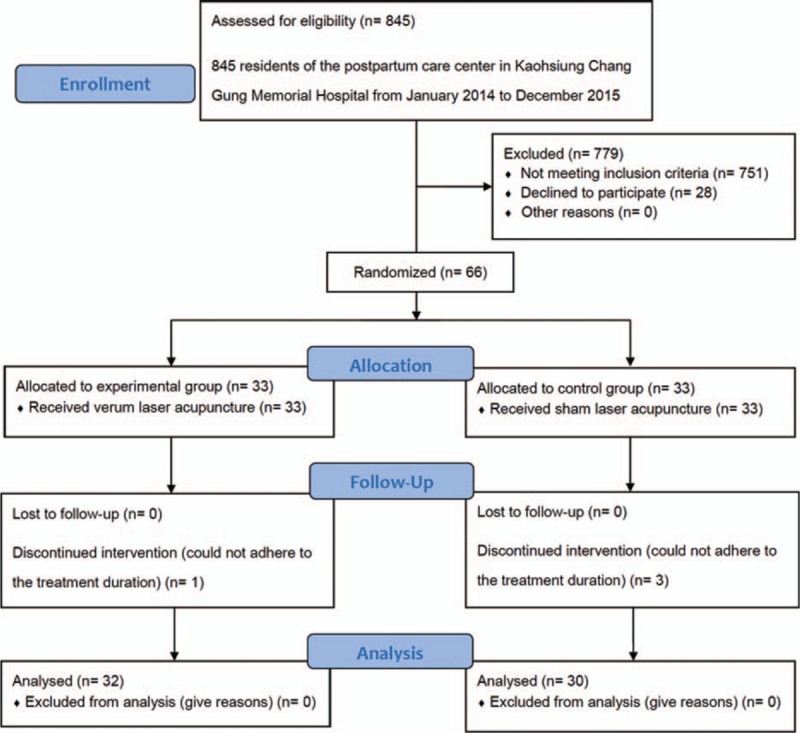
Flowchart of the recruitment and grouping of subjects.

This clinical trial was approved by the Institutional Review Board of Chang Gung Medical Foundation (IRB permit no. 102-2526A3) and registered at ClinicalTrial.gov. (ClinicalTrials.gov ID: NCT02840916). Informed consent was obtained from all subjects before enrollment in the study. After certain initial assessments, a total of 66 participants were enrolled and randomly allocated to either a laser acupuncture group (n = 33) or a control group (n = 33), by using a 1:1 allocation ratio, according to permuted block randomization. The participants were not informed about the different treatment groups and were therefore blinded to the type of intervention applied. The flow diagram of the study procedure is shown in Fig. 1.

The inclusion criteria were as follows: postpartum duration of <1 month, body mass index (BMI) >25, age >20 years, did not use any other medications for weight loss during the study period, and provided informed consent. The exclusion criteria were as follows: presence of a pacemaker; history of seizure or epilepsy; taking immunosuppressant medication; cancer; infectious disease of the skin; taking medications for weight loss, including Chinese herbal preparations, during the study period; receiving other treatment, including supplements, herbs, and medications for weight gain before entering the study; comorbidities (hypothyroidism, renal disease, etc.) that the physician believes would affect the findings; unable to undergo LAT due to other medical conditions; and lack of informed consent.

The cutoffs for overweight (≥23.0 kg/m^2^) and obesity (≥25.0 kg/m^2^) among Asians are lower than the World Health Organization (WHO) criteria.^[3]^ These BMI thresholds differ from those determined for Caucasians because Asians exhibit more comorbidities and a greater fat mass at lower BMI values.^[2,13]^ This study was conducted in Taiwan, and hence, the criteria recommended for Asians were chosen for this analysis.

### 2.2 Study design

This single-blind clinical study was conducted at the postpartum care center in Kaohsiung Chang Gung Memorial Hospital from January 2014 to December 2015. The average duration of admission at our postpartum care center was 18.6 days. Therefore, the treatment duration was set to within 3 weeks. Two laser acupuncture protocols, verum and sham laser acupuncture, were used in the study. Participants underwent a total of 12 LAT sessions, which were performed 5 times a week for approximately 3 weeks. The basic patient characteristics were recorded at baseline, including age, height, prenatal body weight, breastfeeding status, singleton or twin pregnancy, preterm labor, presence of a history of hypertension or diabetes mellitus, and average daily calorie intake. The occurrence of adverse events was recorded over a 3-month period, from the start of LAT to the end of the treatment. If there were any significant adverse events, the treatment was paused or even stopped.

### 2.3 Interventions

#### 2.3.1 Intervention group/laser acupuncture group

**Figure 2:**
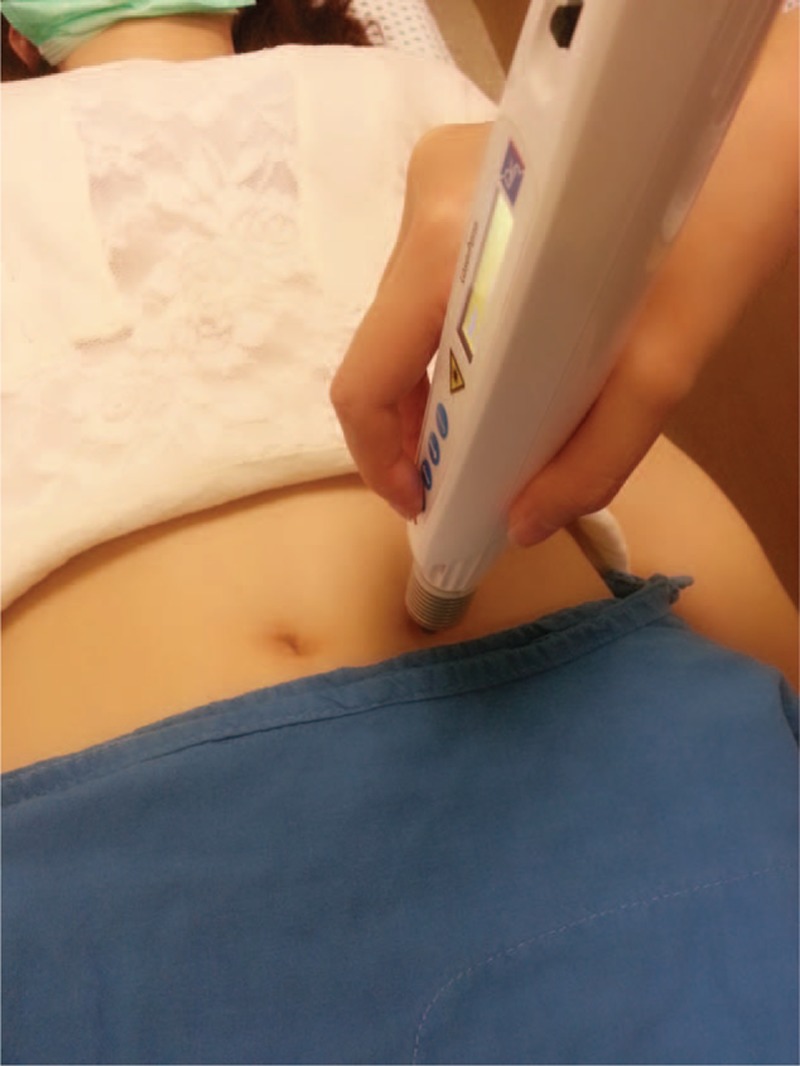
Laser acupuncture at ST25.

The subjects underwent 12 activated laser acupuncture treatment sessions. The sessions were performed 5 times a week, for approximately 3 weeks, by using a gallium aluminum arsenide LaserPen (maximal power, 150 mW; wavelength, 810 nm; area of probe, 0.03 cm^2^; power density, 5 W/cm^2^; pulsed wave; and Bahr frequencies [B1: 599.5 Hz, B2: 1199 Hz, B3: 2398 Hz, B4: 4776 Hz, B5: 9552 Hz, B6: 19,104 Hz, and B7: 38,208 Hz]; RJ-Laser, Reimers & Janssen GmbH, Waldkirch, Germany). The intervention methods including dose and acupoint selection were based on our previous study.^[11]^ We selected the same acupoints in the verum and the sham laser acupuncture group. However, the subjects in the control group also underwent sham laser acupuncture treatment, but without any laser output (no stimulation). The other subjects in the verum group sequentially received 0.375 J of energy at each of the acupoints: stomach and hunger (auricular points; B6 for the right ear and B7 for the left ear), ST25 (Tianshu, B2) (Fig. 2), ST28 (Shuidao, B2), ST40 (Fenglong, B2), SP15 (Daheng, B2), CV9 (Shuifen, B3), and SP6 (Sanyinjiao, B2). The total treatment dose was 5.625* *J/cm^2^. The laser was applied to each point for 5 seconds. All acupoints were selected and localized according to the WHO Standardized Acupuncture Point Location guidelines.^[14]^ In all the subjects, laser application was performed by the same experienced physician who had undergone sufficient training and was a licensed Chinese medicine practitioner in Taiwan.

#### 2.3.2 Control group/sham laser acupuncture group

Subjects in the control group underwent sham laser acupuncture treatment without any laser output. The acupuncture points, application duration, and total number of treatments were similar to those in the laser acupuncture group.

### 2.4 Measurements

Before treatment, an evaluator collected information regarding the patient's age, prenatal weight, average calorie intake, breastfeeding status, full-term birth, normal spontaneous delivery, twin pregnancy, hypertension, and diabetes mellitus. The physician who was involved in treatment application was blinded to the patient characteristics. The primary outcome measure was the change in BMI (weight [kg]/height [m^2^]) from baseline. The secondary outcome measures include body fat percentage (BFP) and circumference of the waist and buttocks. To compare the efficacy of verum LAT with sham laser acupuncture in obese women, we measured the body weight, BFP, and circumference of the waist and buttocks every 6 sessions and during follow-up visits once a month for 3 months. The assessments were performed by an evaluator who was blinded to the intervention details at the time of admission (PRE), after the end of the 12 treatment sessions (POST), and at 3 months after the training was completed (FOLLOW-UP). Thus, both the participants and the evaluators were blinded to the allocated intervention. The waist circumference was measured from the midpoint between the lower margin of the least palpable rib and the top of the iliac crest by using a stretch-resistant tape. The buttocks circumference was measured around the widest portion of the buttocks, with the tape parallel to the floor.^[15]^ Bioelectrical impedance (Omron *handheld device*: HBF-301 Tokyo, Japan.) was used to measure the BFP. The participants’ body weight, height, BFP, waist and buttocks circumference, blood pressure, and heart rate were recorded every 6 sessions until the end of the trial, and the changes in BMI, BFP, and waist-to-buttocks ratio (WBR) were estimated.

### 2.5 Statistical analysis

If we set α = 0.05, β = 0.2, power = 0.8, *P*_c_ = 5%, and *P*_T_ = 30%, the required sample size was determined to be 32.5, as estimated by using the following formula: *f*(α,β) × (*P*_C_[1 − P_C_] + *P*_T_[1 − *P*_T_])/(*P*_T_ − *P*_C_).^[2]^ All data are presented as mean ± standard deviation. Analysis of variance and general linear analysis with repeated measures data were used to evaluate the basic patient characteristics, as well as the change in BMI, BFP, and WBR. Chi-square test and hierarchical log-linear analysis were used for the evaluation of categorical or ordinal variables. Stepwise regression analysis models were used to evaluate BMI, BFP, and WBR. Differences were considered to be statistically significant at a *P* value of <0.05. All analyses were performed with SPSS for Windows, version 17.0 (Statistics 17.0, SPSS, IBM, New York, NY).

## 3 Results

In total, 62 married women (32 in the intervention group and 30 in the control group) completed the therapy program; 4 participants withdrew from the study, as they could not adhere to the treatment duration (Fig. 1). The characteristics of the intervention and control groups did not significantly differ at baseline (Table 1).

**Table 1:**
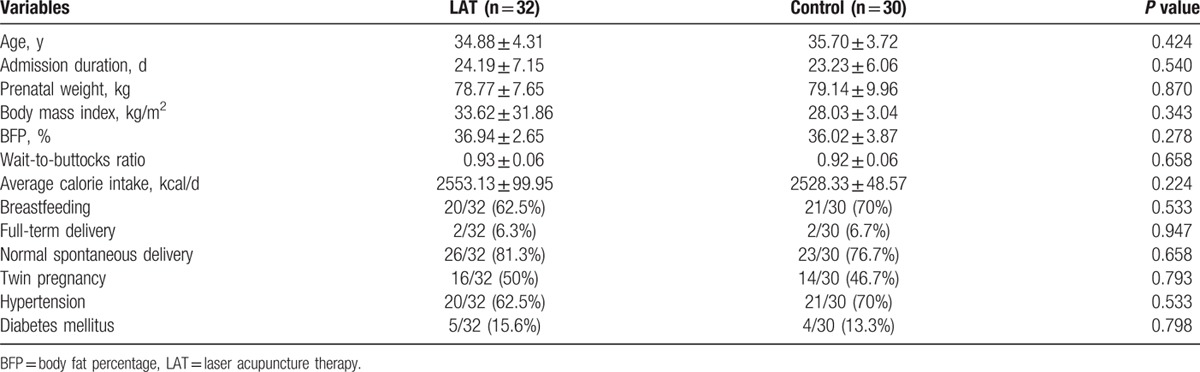
Basic characteristics of women in the postpartum period.

The prenatal body weight was positively correlated with postpartum BMI (β = 0.75, 95% confidence interval [CI] = 0.18–0.27) and BFP (β = 0.55, 95% CI = 0.13–0.29). However, twin pregnancy negatively regressed with postpartum BMI (β = −0.26, 95% CI = −3.72 to −0.95) and BFP (β = −0.25, 95% CI = −3.72 to −0.95). The WBR of women with full-term delivery was higher than that of women with preterm delivery (β = 0.31, 95% CI = 0.01–0.06) (Table 2). There was no difference in the average calorie intake between the LAT group and control group (2553.13 ± 99.95 vs 2528.33 ± 48.57, respectively; *P* = 0.224).

**Table 2:**
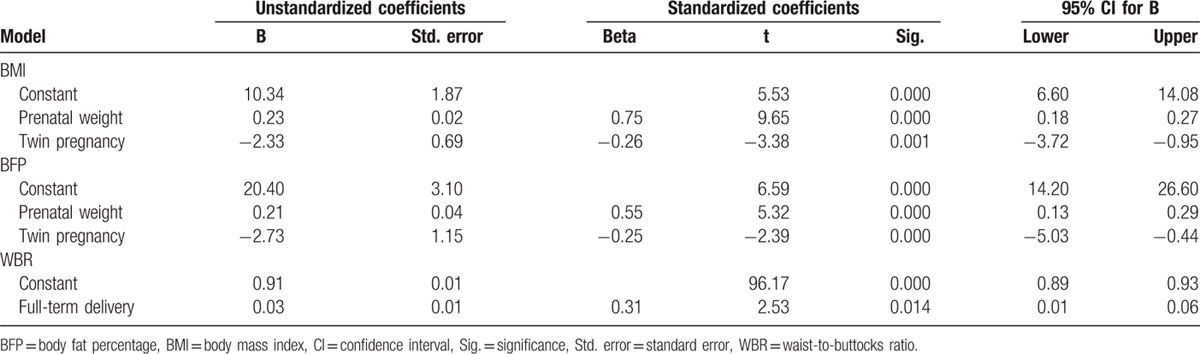
Stepwise regression analysis on BMI, WBR, and BFP of women in the postpartum period.

**Figure 3:**
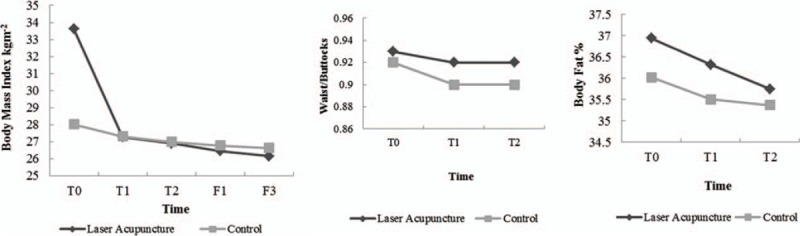
Comparisons of the trends in parameters between the laser acupuncture and control groups. T0: before treatment; T1: after 6 treatment sessions; T2: after 12 treatment sessions; F1: follow-up visit after 1 month; F2: follow-up visit after 3 months.

The analysis of repeated measures data between the laser acupuncture and control groups indicated significant differences in BMI (*P* < 0.001) and BFP (*P* < 0.001) (Table 3). The trends in these parameters are illustrated in Fig. 3. However, no significant difference was noted in the WBR (*P* = 0.09). Moreover, no adverse event was reported during the intervention and follow-up periods.

**Table 3:**
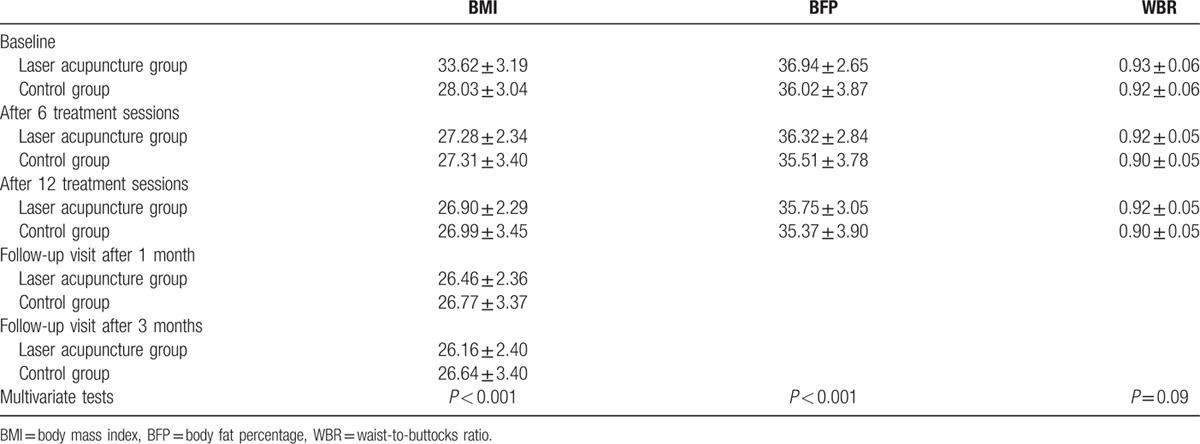
Analysis of repeated measures data on BMI, WBR, and BFP of women in the postpartum period.

## 4 Discussion

Pregnancy is considered as a natural and biological cause of weight recycling and is a period during which women gain substantial weight. In some women, pregnancy markedly alters their future weight-gain trajectory,^[16,17]^ particularly in those women with postpartum weight retention. Our results indicate that laser acupuncture can be effective for postpartum weight control by improving BMI and BFP. Moreover, there was no significant decrease in the daily caloric intake (1.6%) in the present study, which could be due to the postpartum status of the patients.

According to the postpartum regimen followed in Taiwan, most women tend to stay at home or in a postpartum care center during the postpartum period of 1 month; hence, they exhibit decreased physical activity or could have a specific postpartum nourishment diet that may include more meat and fat than usual. Thus, the energy balance may be disturbed. The imbalance between energy intake and expenditure would consequently inhibit weight reduction and even lead to postpartum weight gain.^[18]^ Although a previous review indicated that low-carbohydrate/high-protein diets were as effective as low-fat/high-carbohydrate diets in reducing weight,^[19]^ we did not restrict the food choices of the patients in the present study. Instead, we recorded their daily diet and calculated the average daily caloric intake. No differences in the average daily caloric intake were observed between the intervention and control groups. Moreover, we did not consider the differences in the individual physical activity level as most participants were residing in the same postpartum care center.

Breastfeeding is known to promote postpartum weight loss through the increased caloric expenditure required for lactation^[20]^ or metabolic changes that favor weight loss.^[21]^ Nevertheless, the influence of breastfeeding on weight during the postpartum period is controversial. Although some studies indicated a positive association between breastfeeding and weight loss,^[21–23]^ certain other studies have found no significant association between breastfeeding and postpartum weight loss.^[24,25]^ In the present study, we excluded the effect of breastfeeding as the breastfeeding status did not differ between the intervention and control groups (*P* = 0.533). Moreover, none of the subjects indicated that the intervention disrupted lactation.

Stepwise regression analysis in the present study indicated that prenatal body weight positively regressed and twin pregnancy negatively regressed with postpartum BMI and BFP. Moreover, the WBR of women with full-term delivery was higher than that in women with preterm delivery. However, the prenatal weight, twin pregnancy, and full-term delivery status did not significantly differ between the laser acupuncture and control groups in the present study, and hence their potential impact was excluded. This indicates the advantage of using a randomized control trial design due to its superior analytic power.

Acupuncture is an integral part of TCM and has been used for approximately 3000 years. In its original form, acupuncture is based on the principles of TCM and involves the insertion of fine needles into the skin along the meridians, thus providing a means of altering the flow of energy through the body.^[26]^ Acupuncture attempts to regulate energy balance by stimulating specific acupoints along the meridians and thereby treat the underlying disease. Recently, this technique has become more commonly used as an alternative treatment for obesity. Studies on animals and humans have indicated several potential mechanisms through which acupuncture contributes to weight reduction, including the regulation of obesity-related neuropeptides in the central nervous system,^[27,28]^ as well as the reduction of triglycerides,^[29,30]^ low-density lipoprotein,^[30,31]^ and total cholesterol levels.^[29,30]^

Recently, LAT has been used as a complementary and alternative therapy to traditional acupuncture. LAT is a noninvasive technique involving the stimulation of traditional acupoints by using low-intensity and nonthermal laser irradiation.^[32]^ Compared with needle-based methods for manipulating *Q*_*i*_, LAT has the advantages of being noninvasive and aseptic. Moreover, it is a painless and safe procedure.^[10]^ However, only a few clinical studies have examined the efficacy of laser acupuncture in weight control.^[11,12,33]^ In the present study, the design, including dose and acupoint selection, was based on a previous study performed by Hu et al.^[11]^ In addition, the present study also included a placebo control group and added the SP6 point that influences the metabolism of water. Besides the stimulation of other body acupoints, the stimulation of the hunger and stomach points of the ear is also known to play a role in weight reduction by suppressing ghrelin production.^[34]^

The present study had certain limitations. First, the prepregnancy weight of the patients was not recorded, and hence the postpartum weight trend cannot be assessed. Some studies have indicated that an overweight status before pregnancy and excessive GWG is significantly associated with increased postpartum weight.^[35–37]^ However, another study reported on the possibility of the overestimation of the effect of GWG.^[38]^ Moreover, there is a possibility of recall bias with regard to the prepregnancy BMI and total GWG in the subjects. Second, the average duration of admission in our postpartum care center was 18.6 days, and hence the need to complete the treatment program within 3 weeks was indeed a limitation. Accordingly, the results only indicate the short-term effects of laser acupuncture on postpartum weight control, and further analysis of the long-term effects is needed. Third, the likely presence of some confounders should also be considered. For example, individuals who volunteered for trials were likely to be highly motivated and could modify their medication or increase their exercise during the course of the study. Hence, prepregnancy BMI, educational attainment, psychosocial factors (depressed/anxious or distressed), parity, and total GWG may be possible confounders which could limit the validity of the study findings.^[6–8]^ Furthermore, we only analyzed the BMI during the follow-up period because the data for BFP and WBR were unavailable. As the participants were occupied with childcare, it was difficult to request for a follow-up visit. Although we did conduct telephonic interviews to record some data after the treatment for a period of 3 months, most participants were only able to estimate body weight at home. Therefore, the lack of follow-up for BFP and WBR after 1 and 3 months may also be limitations of this study. In addition, with regard to GWG, first-trimester weight gain may be more strongly associated with maternal weight retention than second- or third-trimester gain. However, further study is needed to evaluate this issue.

In conclusion, we observed that laser acupuncture could reduce postpartum weight retention by improving BMI and BFP, without any impact on the WBR. In particular, positive effects on BMI and BFP were observed following short-term treatment over 3 weeks, which appeared to persist for up to 3 months. As mentioned above, we will determine the long-term effects of this treatment (≥3–6 months) on postpartum weight loss in a future study. Moreover, additional scientific evidence regarding the efficacy of laser acupuncture on obesity should be elucidated based on strong experimental evidence.
